# Causal relationship between gut microflora and dementia: a Mendelian randomization study

**DOI:** 10.3389/fmicb.2023.1306048

**Published:** 2024-01-15

**Authors:** Jinjie Fu, Yuan Qin, Lingyong Xiao, Xiaoyu Dai

**Affiliations:** ^1^Graduate School, Tianjin University of Traditional Chinese Medicine, Tianjin, China; ^2^College of Traditional Chinese Medicine, Tianjin University of Traditional Chinese Medicine, Tianjin, China; ^3^Department of Acupuncture and Moxibustion, First Teaching Hospital of Tianjin University of Traditional Chinese Medicine, Tianjin, China; ^4^National Clinical Research Center for Chinese Medicine Acupuncture and Moxibustion, Tianjin, China

**Keywords:** dementia, gut microflora, Mendelian randomization, causality, Alzheimer’s disease

## Abstract

**Background:**

Numerous pertinent investigations have demonstrated a correlation between gut microflora (GM) and the occurrence of dementia. However, a causal connection between GM and dementia and its subtypes has not yet been clarified.

**Objective:**

To explore the causal association between GM and dementia, including its subtypes, a two-sample Mendelian randomization (TSMR) analysis was used.

**Methods:**

Our data comes from the Genome-Wide Association Study (GWAS). The principal approach employed for the Mendelian randomization study was the inverse-variance weighted method, supplemented by four methods: MR-Egger, weighted median, simple mode, and weighted mode. This was followed by Cochrane’s Q test, MR-Egger intercept test, MR-PRESSO global test, and leave-one-out as sensitivity analysis validation.

**Results:**

Twenty-one GMs associated with any dementia, Alzheimer’s disease, vascular dementia, Lewy body dementia, Parkinson’s disease, and dementia under other disease classifications were derived from the analysis, and 21 passed sensitivity tests.

**Conclusion:**

We confirmed the causal relationship between GM and dementia and its subtypes, derived specific flora associated with increased or decreased risk of dementia, and provided new ideas for preventive, diagnostic, and therapeutic interventions for dementia mediated by gut microbiota.

## 1 Introduction

Dementia is a prevalent neurodegenerative disorder clinically distinguished by cognitive impairment and a gradual deterioration in one’s ability to function autonomously (Liu et al., 2019). According to a WHO report (Cheah et al., 2022), dementia has now become the seventh leading cause of death globally, and it is expected that the number of dementia patients worldwide will reach 139 million by 2050. At the same time, the prevention and treatment of dementia bring a substantial economic and healthcare burden to society and countries, and the global investment in dementia will reach 2.8 trillion dollars by 2030 (World Alzheimer Report, 2015). Alzheimer’s disease represents the prevailing form of dementia, comprising approximately 50 to 70% of cases. Other frequently encountered kinds comprise vascular dementia, Lewy body dementia, Parkinson’s disease, and dementia in other diseases as classified elsewhere (Aarsland, 2020; Wilbur, 2023). To date, the underlying mechanisms leading to dementia have not been clarified, and the medical requirements of individuals with dementia are not well met (Zagórska et al., 2023). Therefore, clarification of dementia-related risk factors, and thus dementia prevention, intervention, and care, can significantly help enhance the well-being and survival rates of individuals with dementia (Livingston et al., 2020).

Gut microbiota (GM) generally refers to bacteria in the human gut. It is involved in regulating a wide range of physiological functions in the host organism and protecting the host from pathogenic bacteria (Álvarez et al., 2021; Kuziel and Rakoff-Nahoum, 2022). Increasingly, GM has been found to fulfill an essential role in the nervous system through the brain-gut axis and has even been implicated in neurodegenerative diseases (Cryan et al., 2020; Mitrea et al., 2022). Studies have demonstrated that GM metabolites, molecules, and endotoxins may affect the central nervous system through the bloodstream or the vagus nerve, affecting brain function and cognitive behavior (Chen et al., 2021). This is undoubtedly a complementary approach to diagnosing and treating dementia, and many scholars have endeavored to address neurodegenerative illnesses through the manipulation of gut microbiota (Möhle et al., 2016; Sasmita, 2019). A systematic evaluation based on dementia studies showed that probiotic supplements improved memory in patients with dementia, as well as elevated levels of brain-derived neurotrophic factor (Ruiz-Gonzalez et al., 2021). In addition, some studies have found differences in GM composition between healthy people and people with cognitive impairment or different types of cognitive impairment, attempting to use this information to make a diagnosis of the disease (Guo et al., 2021; Hung et al., 2022). Therefore, clarifying the influence of different flora on dementia is essential for ascertaining new therapeutic targets for dementia and diagnosing dementia using microbial profiles (Liu et al., 2019; Guo et al., 2021; Cuartero et al., 2023).

Mendelian randomization (MR) is a study method that explores causal relationships between exposure factors and outcomes using single nucleotide polymorphisms (SNPs) as instrumental variables (IVs; Burgess et al., 2017), which is consistent with the principle of random allocation of genetic variation during meiosis, avoiding the influence of confounding variables and the potential for reverse causality (Sekula et al., 2016). This work employed GWAS summary statistics of GW taxa associated with dementia and their subtypes for MR analysis in order to evaluate the risk relationship between genetically determined GW taxa and dementia and its subtypes, which provides evidence for existing findings, new research ideas for pathogenesis that has not yet been clarified, and new directions for the early diagnosis, avoidance, and therapy of all types of dementia.

## 2 Materials and methods

### 2.1 Study design

**Figure 1:**
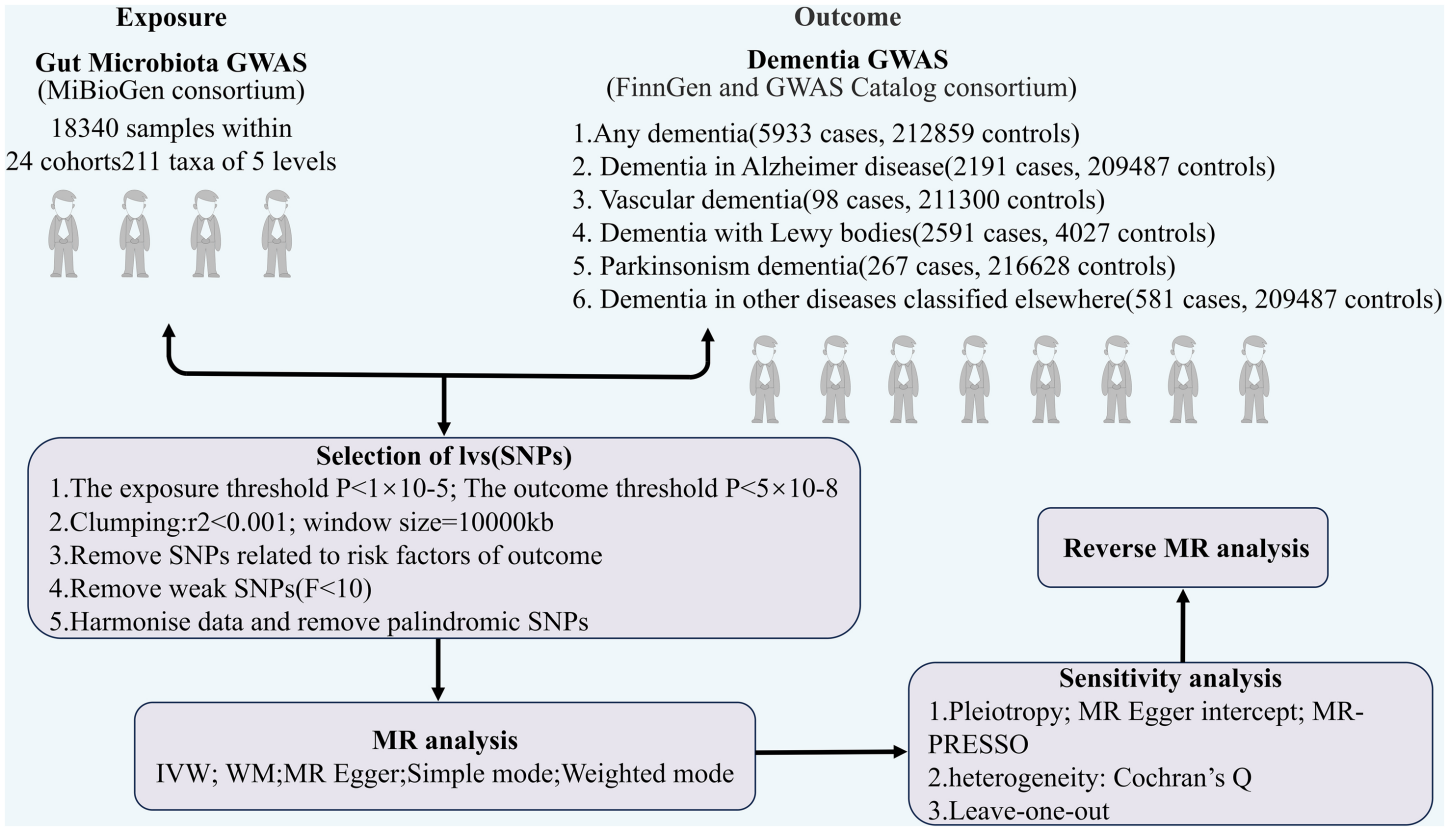
The flowchart of the study.

**Figure 2:**
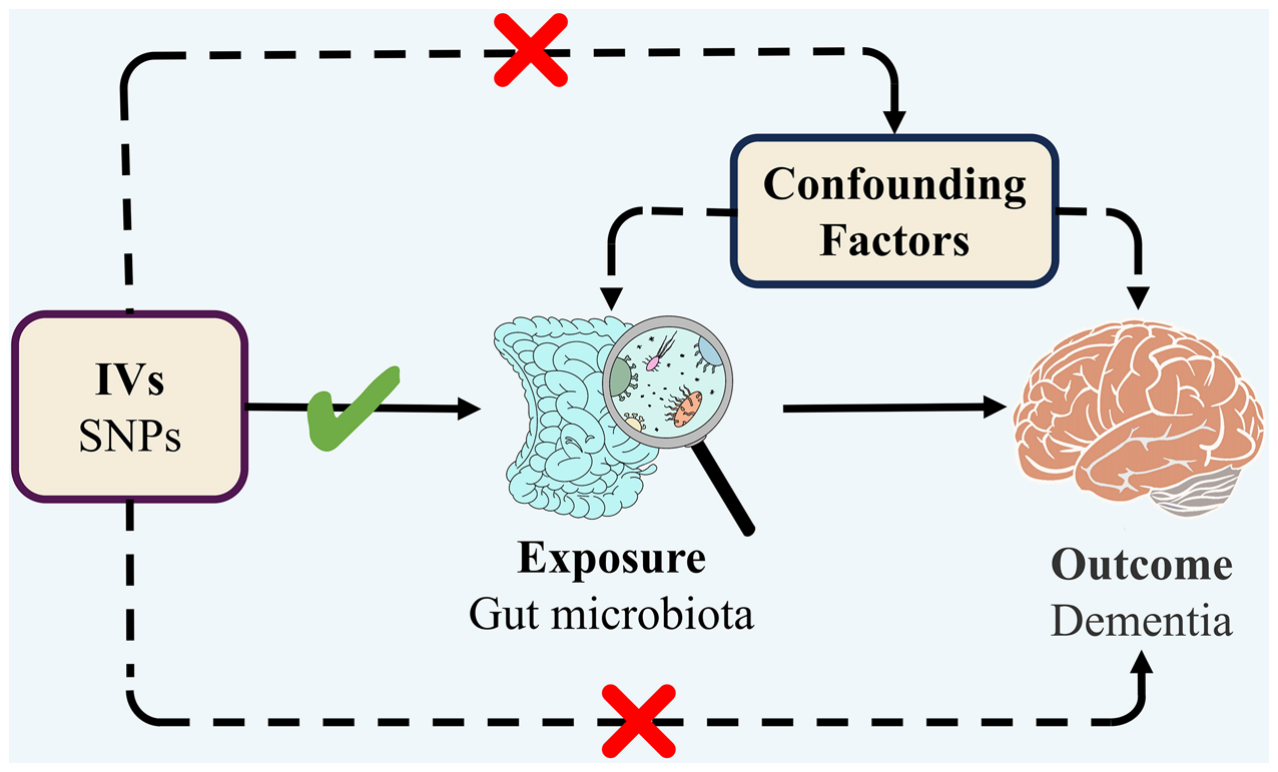
Three assumptions of MR.

TSMR was employed to analyze the association between GM and dementia (any dementia, Alzheimer’s disease, vascular dementia, Lewy body dementia, Parkinson’s disease, dementia in other diseases classified elsewhere) with regard to causality. The overall design of the study is illustrated in Figure 1. In order to carry out a TSMR study, it is imperative that three fundamental assumptions are satisfied: (1) Strong correlation between IVs and exposure; (2) No correlation between IVs and confounders; (3) IVs can only affect outcomes through exposure (EPIC-InterAct Consortium et al., 2015). IVs that fulfill these three assumptions were included in this MR study (Figure 2). This study followed the most updated guidelines (STROBE-MR; Skrivankova et al., 2021).

### 2.2 Sources of data on exposure

The GWAS data for gut microbes were acquired through the MiBioGen consortium^1^ from genomic statistical research by Kurilshikov et al. of 18,340 individuals of European ethnicity from 11 countries (24 cohorts), and the data contained 211 gut microbes with 122,110 variant loci (Kurilshikov et al., 2021). From this GWAS, we screened IVs of gut bacterial taxa in five ranks.

### 2.3 Source of data on endings

GWAS statistics for any dementia, Alzheimer’s disease, vascular dementia, Parkinson’s disease, and dementia in other diseases classified elsewhere were derived from the FinnGen study program.^2^ GWAS statistics for Lewy body dementia were derived from the GWAS Catalog.^3^ The diagnostic standards for dementia are according to F03 in the ICD-10 criteria, where the GWAS dataset has 16,380,466 variant loci from 5,933 patients and 212,859 controls. The diagnostic standards for Alzheimer’s disease are according to G30.901 of the ICD-10 criteria, which contains 16,380,451 variant loci from 2,191 cases and 209,487 controls. The diagnostic standards for Lewy body dementia are according to G31.805 of the ICD-10 criteria, which contains 7,593,175 variant loci from 2,591 cases and 4,027 controls. The diagnostic standards for vascular dementia are according to F01 of the ICD-10 criteria and contain 16,380,453 variant loci from 98 cases and 211,300 controls. The diagnostic standards for Parkinson’s disease are according to the G20 of the ICD-10 criteria and contain 16,380,459 variant loci from 267 cases and 216,628 controls. The diagnostic standards for dementia in other diseases classified elsewhere are according to F02.8 of the ICD-10 criteria and contain 16,380,450 variant loci from 581 cases and 209,487 controls. In addition, three datasets related to any dementia were added as three validation groups (Table 1).

**Table 1:** Details of the datasets included in this study.

	Trait	Year	Sex	Population	Case	Control	Number of SNPs	PMID/URL (Data download)
Exposure	Any dementia	2021	Male and female	European	5,933	212,859	16,380,463	https://gwas.mrcieu.ac.uk/datasets/finn-b-KRA_PSY_DEMENTIA/
	Alzheimer’s disease	2021	Male and female	European	2,191	209,487	16,380,451	https://gwas.mrcieu.ac.uk/datasets/finn-b-F5_ALZHDEMENT/
	Vascular dementia	2021	Male and female	European	98	211,300	16,380,453	https://gwas.mrcieu.ac.uk/datasets/finn-b-VD_MX/
	Lewy body dementia	2021	Male and female	European	2,591	4,027	7,593,175	33,589,841
	Parkinson’s disease	2021	Male and female	European	267	216,628	16,380,459	https://gwas.mrcieu.ac.uk/datasets/finn-b-PD_DEMENTIA/
	Dementia in other diseases classified elsewhere	2021	Male and female	European	581	209,487	16,380,463	https://gwas.mrcieu.ac.uk/datasets/finn-b-F5_DEMINOTH/
Outcome	Gut microbe	2021	Male and female	European	-	-	122,110	33,462,485
Validation group	Any dementia	2021	Male and female	European	5,933	166,584	16,380,199	https://gwas.mrcieu.ac.uk/datasets/finn-b-KRA_PSY_DEMENTIA_EXMORE/
	Any dementia	2021	Male and female	European	7,284	209,487	16,380,450	https://gwas.mrcieu.ac.uk/datasets/finn-b-F5_DEMENTIA/
	Any dementia	2021	Male and female	European	7,395	211,397	16,380,465	https://gwas.mrcieu.ac.uk/datasets/finn-b-F5_DEMENTIA_INCLAVO/

### 2.4 Selection of IVs

We screened the relevant IVs according to the following standards: (1) a significant threshold (*p* < 5 × 10^−8^) for IVs was associated with exposure and outcome, but the quantity of eligible IVs (exposure) was low, so a more appropriate threshold (*p* < 1 × 10^−5^) was used to acquire a larger quantity of IVs (Lv et al., 2021; Zeng et al., 2023); (2) the chain imbalance coefficient r^2^ < 0.001, distance = 10,000 kb was set to remove the presence of chain imbalance among IVs; (3) to avoid the effects of horizontal pleiotropy, IVs linked to dangerous elements of dementia were eliminated by the utilization of PhenoScanner (Kamat et al., 2019); (4) palindromic SNPs were removed from IVs; and (5) to avoid bias from weak instrumental variables, we removed IVs with *F* < 10 (Burgess et al., 2011).

### 2.5 Statistical analysis

In TSMR analysis of gut microbes and dementia (any dementia, Alzheimer’s disease, vascular dementia, Lewy body dementia, Parkinson’s disease, dementia in other diseases classified elsewhere) in causality, the fixed-effects IVW method and the random-effects IVW method were the main methods (Burgess et al., 2013). The choice between the two is determined by the heterogeneity between IVs, if there is heterogeneity in Cochrane’s Q test (*p* < 0.05), then random-effects IVW method was used, otherwise fixed-effects IVW method or random-effects IVW method was used. Therefore, in this TSMR analysis, We chose the random-effects IVW method as the main method (Greco et al., 2015). In addition, MR-Egger, weighted median, simple mode, and weighted mode can complement IVW (Bowden et al., 2015, 2016), and ORs and 95% confidence intervals were also obtained. A causal relationship between gut microbes and dementia was considered likely if the outcome of one TSMR method was remarkable (*p* < 0.05; Jin et al., 2023), and the causal relationship was considered reliable if the results of two or more TSMR methods were significant (Ni et al., 2021).

Sensitivity analyses took place to verify the robustness of the findings, and Cochrane’s Q test was used to test for heterogeneity. The IV was considered heterogeneous if *p* < 0.05. The MR-Egger method’s intercept term indicates horizontal multiplicity in the IVs, and if this intercept term is significantly different from 0, it indicates the presence of horizontal multiplicity (Burgess and Thompson, 2017). MR-PRESSO is also commonly used to test for horizontal multiplicity (Verbanck et al., 2018). Finally, the validation of the data was conducted using the leave-one-out procedure (Xiang et al., 2021). The investigation was carried out utilizing R program (version 4.3.0). The “Two SampleMR” R package^4^ and “MRPRESSO” R package^5^ were used for our MR study.

### 2.6 Reverse MR analysis

Assuming that there are relevant GMs that can have an effect on dementia and its subtypes in the final findings, we will further conduct a reverse MR analysis to explore the effect of dementia on GMs, with dementia as the exposure and GMs as the outcome to avoid reverse causality interfering with the results of this study.

## 3 Results

### 3.1 IV details

After screening the above entries, 605 IVs associated with dementia were finally obtained, involving 60 GMs. Detailed information can be found in Supplementary Table 1. All IVs involved had *F* values greater than 10 (range 16.91–85.37), so there were no weak instrumental variables. These IVs were categorized into five classes: phylum, class, order, family, and genus, comprising two phylum (14 IVs), four classes (40 IVs), nine orders (90 IVs), 13 families (134 IVs), and 32 genera (327 IVs). Because of the inclusionary relationship between gut microbial classifications, there may be a substantial overlap of SNPs and their associated orders contained in various types of enterobacteria.

### 3.2 Results of the TSMR analysis

**Figure 3:**
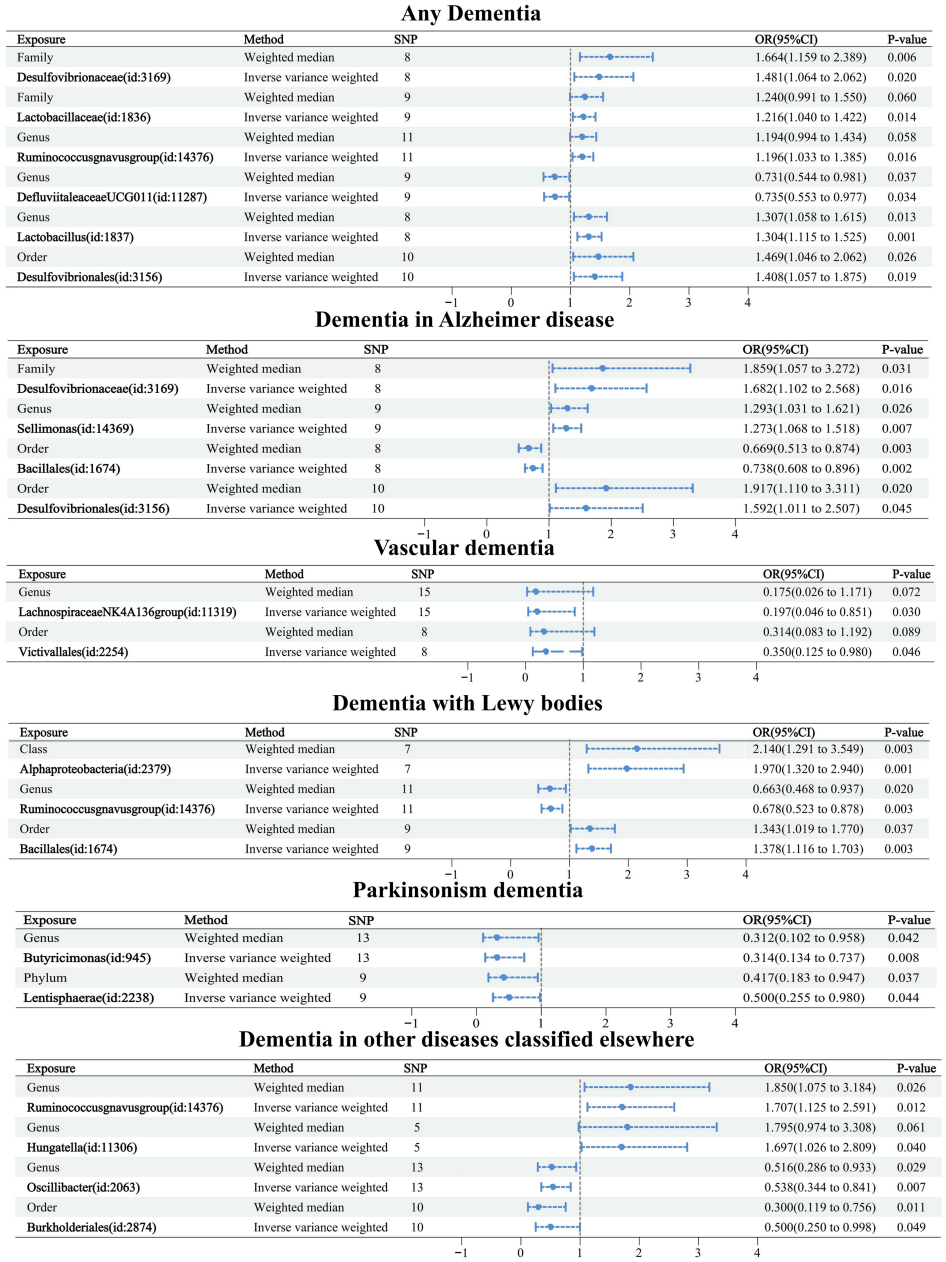
Forest plot of causal relationships between 21 GMs and dementia under cross-validation.

Causal relationships between the 60 GMs screened and dementia were analyzed using five TSMR methods: IVW, MR-Egger, weighted median, simple mode, and weighted mode (Supplementary Table 2). Potential causal relationships between the 60 GMs and dementia were determined using two TSMR methods, in which six GMs associated with dementia, four GMs associated with Alzheimer’s disease, two GMs associated with vascular dementia, three GMs associated with Lewy body dementia, two GMs associated with Parkinson’s disease, and four GMs associated with other diseases under the classification of dementia-associated GMs, and cross-validation was performed (Table 2; Figure 3). Our attention was directed toward the 21 causal associations that have a relatively steady nature.

Causal relationships were obtained for six related GMs in any dementia using the IVW method, and all six relationships were more stable under IVW and WM cross-validation. Among them, family Desulfovibrionaceae (OR: 1.481, 95% confidence interval (CI): 1.064–2.062, *p* = 0.020), family Lactobacillaceae (OR: 1.216. CI: 1.040–1.422, *p* = 0.014), genus Ruminococcusgnavus group (OR: 1.196, CI: 1.033–1.385, *p* = 0.016), genus Lactobacillus (OR: 1.304, CI: 1.115–1.525, *p* = 0.001), and order Desulfovibrionales (OR: 1.408, CI: 1.057–1.875, *p* = 0.019) were related to increased risk of dementia. Genus Defluviitaleaceae UCG011 (OR: 0.735, CI: 0.553–0.977, *p* = 0.034) was related to a reduced risk of dementia.

**Table 2:** Causal relationship between intestinal flora and dementia and its classified diseases.

Outcome	Exposure	Method	SNPs	*p*-value	OR	95%CI
Any dementia	Family Desulfovibrionaceae (id:3169)	WM	8	0.006	1.664	1.159–2.389
		IVW		0.02	1.481	1.064–2.062
	Family Lactobacillaceae (id:1836)	WM	9	0.06	1.240	0.991–1.550
		IVW		0.014	1.216	1.040–1.442
	Genus Ruminococcusgnavus group (id:14376)	WM	11	0.058	1.194	0.994–1.434
		IVW		0.016	1.196	1.033–1.385
	Genus Defluviitaleaceae UCG011 (id:11287)	WM	9	0.037	0.731	0.544–0.981
		IVW		0.034	0.735	0.553–0.977
	Genus Lactobacillus (id:1837)	WM	8	0.013	1.307	1.058–1.615
		IVW		0.001	1.304	1.115–1.525
	Order Desulfovibrionales (id:3156)	WM	10	0.026	1.469	1.046–2.062
		IVW		0.019	1.408	1.057–1.875
Alzheimer’s disease	Family Desulfovibrionaceae (id:3169)	WM	8	0.031	1.057	1.859–3.272
		IVW		0.016	1.102	1.682–2.568
	Genus Sellimonas (id:14369)	WM	9	0.026	1.031	1.293–1.621
		IVW		0.007	1.068	1.273–1.518
	Order Bacillales (id:1674)	WM	8	0.003	0.513	0.669–0.874
		IVW		0.002	0.608	0.738–0.896
	Order Desulfovibrionales (id:3156)	WM	10	0.02	1.110	1.917–3.311
		IVW		0.045	1.011	1.592–2.507
Vascular dementia	Genus Lachnospiraceae NK4A136 group (id:11319)	WM	15	0.072	0.026	0.171–1.175
		IVW		0.03	0.046	0.197–0.851
	Order Victivallales (id:2254)	WM	8	0.089	0.083	0.314–1.192
		IVW		0.046	0.125	0.350–0.980
Lewy body dementia	Class Alphaproteobacteria (id:2379)	WM	7	0.003	1.291	2.140–3.549
		IVW		0.001	1.320	1.970–2.940
	Genus Ruminococcusgnavus group (id:14376)	WM	11	0.02	0.468	0.663–0.937
		IVW		0.003	0.523	0.678–0.878
	Order Bacillales (id:1674)	WM	9	0.037	1.019	1.343–1.770
		IVW		0.003	1.116	1.378–1.703
Parkinson’s disease	Genus Butyricimonas (id:945)	WM	13	0.042	0.102	0.312–0.958
		IVW		0.008	0.134	0.314–0.737
	Phylum Lentisphaerae (id:2238)	WM	9	0.037	0.183	0.417–0.947
		IVW		0.044	0.255	0.500–0.980
Dementia in other diseases classified elsewhere	Genus Ruminococcusgnavus group (id:14376)	WM	11	0.026	1.075	1.850–3.184
		IVW		0.012	1.125	1.707–2.591
	Genus Hungatella (id:11306)	WM	5	0.061	0.974	1.795–3.308
		IVW		0.04	1.026	1.697–2.809
	Genus Oscillibacter (id:2063)	WM	13	0.029	0.286	0.516–0.933
		IVW		0.007	0.344	0.538–0.841
	Order Burkholderiales (id:2874)	WM	10	0.011	0.119	0.3–0.756
		IVW		0.049	0.250	0.5–0.998

Causal relationships were obtained for nine related GMs in Alzheimer’s disease using the IVW method, and four relationships were more stable under IVW and WM cross-validation. Among them, family Desulfovibrionaceae (OR: 1.682, CI: 1.102–2.568, *p* = 0.016), genus Sellimonas (OR: 1.273, CI: 1.068–1.518, *p* = 0.007), and order Desulfovibrionales (OR: 1.592, CI: 1.011–2.507, *p* = 0.045) were associated with increased risk of Alzheimer’s disease. Order Bacillales (OR: 0.738, CI: 0.608–0.896, *p* = 0.002) was related to a decreased risk of Alzheimer’s disease.

Causal relationships were obtained for 14 relevant GMs in vascular dementia using the IVW method, and two relationships were more stable under IVW and WM cross-validation. Among them, genus Lachnospiraceae NK4A136 group (OR: 0.197, CI: 0.046–0.851, *p* = 0.030) and order Victivallales (OR: 0.350, CI: 0.125–0.980, *p* = 0.030) were related to a reduced risk of vascular dementia.

Causal relationships were obtained for nine relevant GMs in Lewy body dementia using the IVW method, and three relationships were more stable under IVW and WM cross-validation. Among them, class Alphaproteobacteria (OR: 1.970, CI: 1.320–2.940, *p* = 0.001) and order Bacillales (OR: 1.378, CI: 1.116–1.703, *p* = 0.030) were associated with increased risk of Lewy body dementia. Genus Ruminococcusgnavus group (OR: 0.678, CI: 0.523–0.878, *p* = 0.003) was related to a reduced risk of Lewy body dementia.

Causal relationships were obtained for 10 related GMs in Parkinson’s disease using the IVW method, and two relationships were more stable under IVW and WM cross-validation. Among them, genus Butyricimonas (OR: 0.314, CI: 0.134–0.737, *p* = 0.008) and phylum Lentisphaerae (OR: 0.500, CI: 0.255–0.980, *p* = 0.044) were related to a reduced risk of Parkinson’s disease.

Causal relationships were obtained for 12 relevant GMs in dementia in other diseases classified elsewhere using the IVW method, and four relationships were more stable under IVW and WM cross-validation. Among them, genus Ruminococcusgnavus group (OR: 1.707, CI: 1.125–2.591, *p* = 0.012) and genus Hungatella (OR: 1.697, CI: 1.026–2.809, *p* = 0.040) were associated with an increased risk of dementia in other diseases classified elsewhere. Order Burkholderiales (OR: 0.500, CI: 0.250–0.998, *p* = 0.049) and genus Oscillibacter (OR: 0.538, CI: 0.344–0.841, *p* = 0.007) were related to decreased risk of dementia in other diseases classified elsewhere.

**Figure 4:**
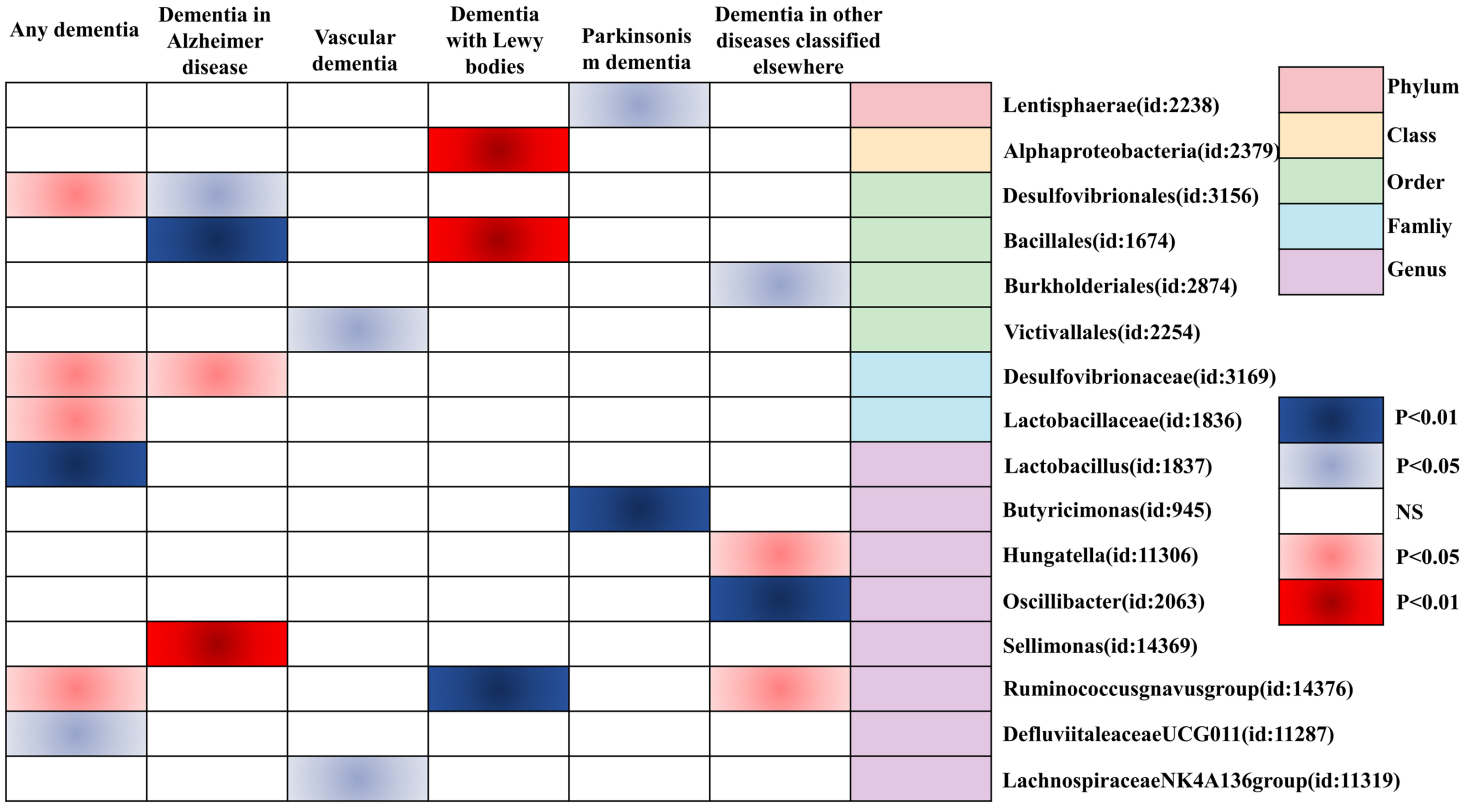
Heatmap of GM causally associated with dementia identified by the IVW method. Red represents risk factors, and blue represents protective factors.

Finally, we utilized a heat map to causally present the results of the study in the form of various types of GMs and any dementia, Alzheimer’s disease, vascular dementia, Lewy body dementia, Parkinson’s disease, and dementia in other diseases classified elsewhere (Figure 4).

### 3.3 Sensitivity analysis

Cochrane’s Q test showed no heterogeneity among the 20 colonies except for the genus Defluviitaleaceae UCG011 (*p* = 0.031) in dementia, which had a value of *p* greater than 0.05 (Supplementary Table 3). Genus Defluviitaleaceae UCG011 had *p* < 0.05 (*p* = 0.002) in the fixed-effects IVW model, suggesting the presence of causality, and also p < 0.05 (*p* = 0.034), OR: 0.735, CI: 0.553–0.977 in the random-effects IVW model, and a cause-and-effect link was also present. The MR-Egger regression intercepts for the 21 GMs showed no horizontal pleiotropy, with *p*-values greater than 0.05 (Supplementary Table 4). The MR-PRESSO Global test value of *p* > 0.05 also demonstrated no horizontal pleiotropy (Supplementary Table 4). Leave-one-out results showed that phasing out any of the SNPs did not affect the overall results, so this MR analysis has good robustness (Supplementary Figure S1).

### 3.4 Reverse MR analysis results

**Figure 5:**
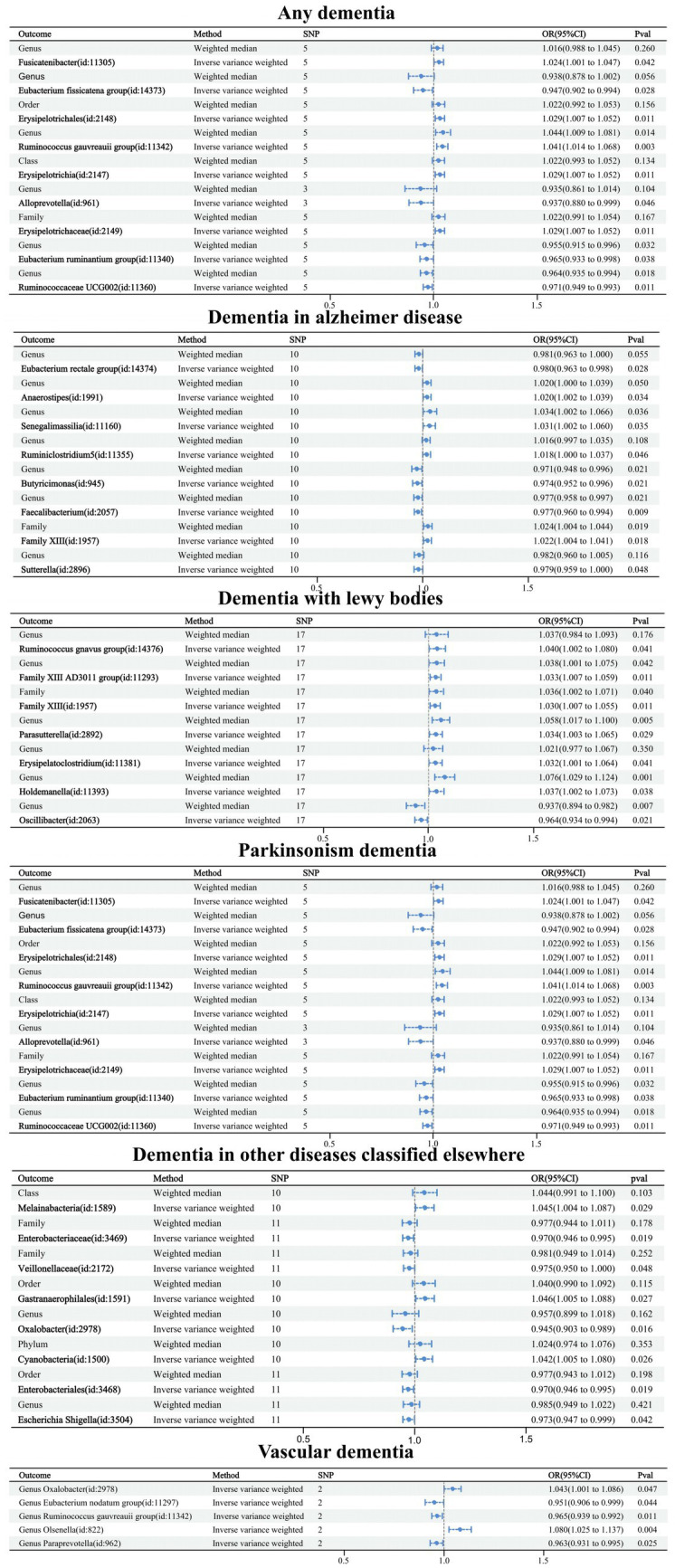
Forest plot of causal relationships between dementia and 50 GMs under cross-validation.

Out of the 211 GMs, a total of 50 GMs affected by dementia and its subtypes were finally obtained, including 13 GMs affected by overall dementia, eight GMs by Alzheimer’s disease, five GMs by vascular dementia, seven GMs by Lewy body dementia, nine GMs by Parkinson’s disease, and eight GMs by dementia under the classification of other diseases. Five TSMR methods—IVW, MR-Egger, weighted median, simple mode, and weighted mode—were used to analyze the causal relationships between the different types of dementia and the 50 GMs (Supplementary Table 5). Forest plots were drawn using IVW and WM cross-validation (Figure 5). Upon comparison with the positive MR results, it was found that among the 21 GMs we focused on for causality with dementia, there was only a reverse causality between Lewy body dementia and genus Ruminococcusgnavus group (id: 14376), and no reverse causality was found between the remaining 20 GMs and dementia. Further sensitivity analysis of MR results between Lewy body dementia and genus Ruminococcusgnavus group (Table 3) was performed, and the test showed no heterogeneity or horizontal pleiotropy in this result.

**Table 3:** Sensitivity test of DLB with genus Ruminococcusgnavus group.

Exposure	Outcome	Method	Q	Value of *p*	MR-Egger intercept test			MR-PRESSO global test	
					Egger-intercept	SE	Value of *p*	RSS obs	Value of *p*
Lewy body dementia	Genus Ruminococcusgnavus group	MR-Egger	14.264	0.506	0.003	0.010	0.792	15.438	0.635
		IVW	14.336	0.574					

### 3.5 Validation group MR analysis results

**Figure 6:**
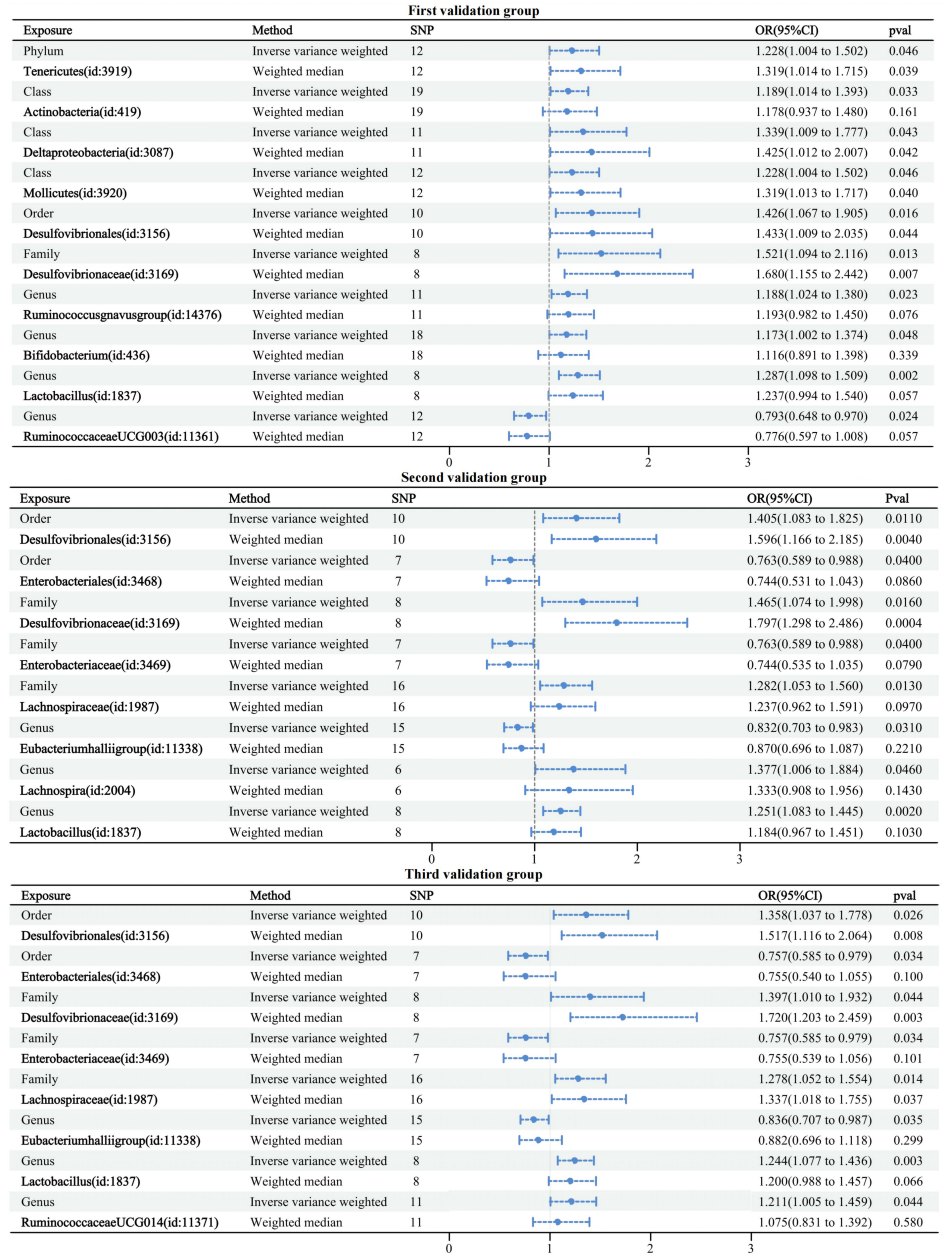
Forest plot of causal relationships between GMs and three validation groups under cross-validation.

The first validation dataset obtained 121 IVs of GMs associated with dementia involving 10 GMs; the second obtained 77 IVs of GMs associated with dementia involving eight GMs; and the third obtained 82 IVs of GMs associated with dementia involving eight GMs. The MR analysis methodology was consistent with the above studies, and detailed information on the results can be found in Supplementary Table 6. All results passed sensitivity tests. Similarly, a forest plot of the IVW and WM cross-tests was plotted and is shown in Figure 6. Compared to the six GMs associated with the formal group any dementia, five overlapping GMs were in the first validation group and four in the second and third validation groups. Three validation groups had four GMs overlapping with the formal group any dementia, accounting for 66.7% of the formal any dementia group, 40% of the first validation group, and 50% of the second and third validation groups. Therefore, the selected formal group dataset is representative.

## 4 Discussion

In this study, by combining MR analysis and sensitivity analysis, 21 GMs were identified as being causally associated with dementia (any dementia, Alzheimer’s disease, vascular dementia, Lewy body dementia, Parkinson’s disease, and dementia in other diseases classified elsewhere). Among them, Desulfovibrionaceae, Lactobacillaceae, Ruminococcusgnavus group, Lactobacillus, Desulfovibrionales, Sellimonas, Bacillales, and Hungatella were positively associated with the risk of outcome disease, and therefore, there may be a risk for the corresponding types of dementia. Defluviitaleaceae UCG011, Bacillales, Lachnospiraceae NK4A136 group, Victivallales, Alphaproteobacteria, Ruminococcusgnavus group, Butyricimonas, Lentisphaerae, Oscillibacter, and Burkholderiales were negatively correlated with the risk of outcome disease, and they may be protective against the corresponding types of dementia.

Understanding the pathogenesis of dementia and the role GM plays in this process is critical to preventing and intervening in dementia. The gut-brain axis is the pathway of communicating among the nervous system and the gastrointestinal tract, which mainly includes the central nervous system (CNS), enteric nervous system (ENS), hypothalamic–pituitary–adrenal axis (HPA), and autonomic nervous system (ANS; Cryan et al., 2019). Moreover, it has been found that GM can influence the pathophysiological processes of diseases such as Alzheimer’s disease and Parkinson’s through ENS (Glinert et al., 2022). For example, it activates the ENS and uses the vagus nerve as a pathway to communicate with the brain (Wang et al., 2020). In addition, GM triggers the progression of a metabolic, inflammatory response that promotes neuroinflammation by engaging in processes that disrupt the blood–brain barrier (BBB), activating astrocytes and microglia, and leading to the deposition of β-amyloid (Aβ), which is now recognized as a significant contributing factor in neurodegenerative diseases (Cryan et al., 2019; Wei et al., 2020; Mou et al., 2022). Relevant scholars have proven that the majority of the variable risk factors for dementia are associated with GM alterations by studying the different variable risk factors for each type of dementia and the different roles of GM for each factor (Cabrera et al., 2021). However, the constitution of GM is subject to the effect of numerous causes, and the diversity of GM may vary due to inconsistencies in gender, ethnicity, and environment.

In our study, we found that order Desulfovibrionales (OR: 1.592, CI: 1.011–2.507, *p* = 0.045) and family Desulfovibrionaceae (OR: 1.682, CI: 1.102–2.568, *p* = 0.016) were strongly related to a high risk of Alzheimer’s disease. The results of related experiments showed that Desulfovibrionaceae abundance at the family and genus levels was significantly higher in amyloid precursor protein transgenic mice than in wild mice (Shen et al., 2017). Abnormal production and processing of Aβ and hyperphosphorylation of tau proteins are the molecular signatures of Alzheimer’s disease (He et al., 2020). GM has been shown to reduce Aβ load in patients with Alzheimer’s disease (Li et al., 2019), and related researchers have found that brain Aβ accumulation is negatively correlated with the family Desulfovibrionaceae (Sheng et al., 2022). *Tetragonia Tetragonioides* Kuntze (TTK) ameliorates memory by decreasing Aβ deposition and modulating GM, with more Desulfovibrionales in the AD-Control group than AD-TTK (Kim et al., 2020). The above studies mentioned the family/order Desulfovibrionales as clinically significant for Alzheimer’s disease. However, the results of the two studies on Aβ deposition in the brain conflicted. In the present study, we found with MR analysis that the family/order Desulfovibrionaceae was associated with an increased risk of developing dementia and Alzheimer’s disease. A growing body of research has been able to demonstrate that altering GMs can attenuate microglia-mediated neuroinflammation and reduce Aβ deposition in the brain, thereby improving cognition (Abraham et al., 2019; Sun et al., 2020; Benichou Haziot and Birak, 2023). Supporting the above findings, we propose that the effect of family/order Desulfovibrionaceae on patients of dementia or Alzheimer’s disease might be related to brain Aβ deposition. However, the exact mechanism of action has not yet been confirmed. Inhibition of patient-specific family/order Desulfovibrionaceae and further study of its pathogenesis based on this may become a new way of intervention to prevent or delay Alzheimer’s disease.

Probiotics are non-pathogenic microorganisms and are beneficial to the organism’s health, with a great capacity to rebuild the microbiota and restore health (Den et al., 2020). Notably, probiotic treatment attenuates age-related learning and memory deficits by reducing microglia activation (Go et al., 2021). Therefore, it has been used as a potential treatment to alleviate psychiatric disorders, including cognitive impairment (CI; Azad et al., 2018). Lactobacillales as a probiotic has been widely used in various CI-related studies, and a study was conducted to induce the expression of brain-derived neurotropic factor (BDNF), inhibit NF-κB activation, and regulate GM in mice to alleviate CI accompanied by systemic inflammation through Lactobacillus griseus (Yun et al., 2023). A systematic evaluation showed increased levels of brain-derived neurotrophic factor, improved inflammatory profile, and cellular biomarker modulation in patients with dementia taking probiotic Lactobacillus (Ruiz-Gonzalez et al., 2021). In addition, the AD-Control group with excessive brain Aβ deposition decreased in the order Lactobacillales (Lactobacillales) compared to the AD-TTK group (Kim et al., 2020). A Mediterranean diet (MeDi) containing very high amounts of Lactobacillales has also been highly effective in preventing Alzheimer’s disease (Trichopoulou and Lagiou, 1997; Walker, 2000; Alfawaz and Aljumah, 2012). It is believed that the evidence that Lactobacillus reduces blood ammonia levels not only offers a connection between Alzheimer’s disease and the MeDi but also lays the groundwork for hyperammonemia and the pharmacology of various neurological disorders (Alfawaz and Aljumah, 2012; Jin et al., 2018). The above research demonstrated the protective function of Lactobacillales in CI from different angles of action. However, our results showed that the family Lactobacillaceae and genus Lactobacillus were weakly correlated with the increased risk of dementia. The reason may be related to sample size, genetics, and research scope.

In addition, our results also showed causal associations with outcomes for probiotics, including Defluvititaleaceae UCG011 associated with dementia, Bacillale associated with Alzheimer’s disease, Ruminococcusgnavus group associated with Lewy body dementia, Lachnospiraceae NK4A136 group and Victivallales strongly associated with vascular dementia, Butyricimonas and Lentisphaerae strongly associated with Parkinson’s disease, and Oscillibacter and Burkholderiales strongly associated with dementia in other diseases classified elsewhere. Some of these results are consistent with existing research findings where Bacillussubtilis was shown to have a protective effect on neurons and behavior in the *Caenorhabditis elegans* AD model and can help alleviate Alzheimer’s disease (Cogliati et al., 2020). Butyricimona has also been shown to be strongly associated with the reduced hippocampal volume associated with cognitive disorder. Jang hypothesized that acupuncture alleviated inflammation in mice with Parkinson’s disease due to an increase in Butyricimonas (Jang et al., 2020; Liang et al., 2022). Neoagarotetraose (NAT) was shown to modulate GM and thereby attenuate brain damage in mice with Alzheimer’s disease, with a remarkable rise of intestinal bacterial genera (Lactobacillus, Butyricimonas, and Akkermansi) observed after NAT treatment (Li et al., 2023). Our study clarified the beneficial bacterial genera for dementia, Alzheimer’s disease, Parkinson’s disease, and Lewy body dementia. This might be a novel research line for the clinical therapy of various types of dementia.

Short-chain fatty acids (SCFAs), which mainly include acetate, propionate, and butyrate, are metabolites produced by GM. Butyrate in SCFAs has anti-inflammatory effects (Mirzaei et al., 2021) and can improve cognitive function by mediating inflammatory responses and inducing Aβ phagocytosis in microglia (Xie et al., 2023). It has been found that Alzheimer’s disease may occur when butyrate is deficient (Tran et al., 2019). Interestingly, propionate induced higher levels of microglia activation than butyrate (Hou et al., 2021), and this hyperactivated state may reduce their ability to phagocytose Aβ, which may have a differential effect on the disease (Xie et al., 2021). When excessive propionate is ingested, there is an increased risk of developing Alzheimer’s disease (Killingsworth et al., 2021). Ruminococcaceae can promote the production of SCFAs and can be associated with diseases of cognitive dysfunction by affecting the expression of proteins involved in neurotransmission (D’Amato et al., 2020). This study showed that the Ruminococcusgnavus group was associated with a risk of dementia, Lewy body dementia, and dementia in other diseases classified elsewhere. However, its high and low risk of different outcome diseases was inconsistent, and we hypothesized that this might be related to the metabolite SCFAs it produces. The different types and doses of SCFAs might be the influencing factors. In addition, the results of the reverse MR analysis done in this study suggested that elevated levels of Ruminococcusgnavus group were associated with an increased risk of Lewy body dementia. Therefore, the present study provides possible mechanism points of SCFAs for dementia at the microbial level, and its specific role and association need to be further explored.

In addition, this study found a strong risk association between Alphaproteobacteria and Lewy body dementia [OR = 1.97 (95% CI: 1.320–2.940) *p* = 0.001]. It has been shown that GM is associated with Lewy body dementia, a pathology of dementia characterized by aggregation of α-synuclein, in which the microbe-gut-brain axis plays a vital role through a variety of potential mechanisms (Ryman et al., 2023). However, research into the relationship between Alphaproteobacteria and Lewy body dementia is scarce; Alphaproteobacteria is usually associated with depression, and antidepressants can reduce their abundance (Lukić et al., 2019). Therefore, the conclusion of this study provides suggestions for future research areas with regard to Alphaproteobacteria for the treatment of Lewy body dementia, which may be the key mechanism of its pathogenesis or a potential therapeutic target.

Current research on GM and various types of dementia is both a hot topic and a great challenge at the same time. Since there is no method of preventing, reversing, or eradicating Alzheimer’s disease, medications licensed for the therapy of Alzheimer’s disease have only been able to slow progression to improve symptoms (Breijyeh and Karaman, 2020). Therefore, in terms of GM and dementia, future research should focus on identifying specific GM bacteria with the pathogenesis of dementia. On the one hand, different GM taxa may have diagnostic value for various types of dementia. On the other hand, the risk of dementia can be reduced through the development of new drugs, disease prevention, treatment, and other aspects.

The limitations of this study are as follows: (i) since the number of IVs satisfying the strict threshold (*p* < 5 × 10^−8^) was minimal, a relatively loose threshold (*p* < 1 × 10^−5^) was used to screen the IVs; (ii) in this study, part of the data for dementia was obtained in 2021 from the FinnGen database version R5, the most recent online data for the IEU data. Nevertheless, there are still limitations regarding the duration of data collection and the quantity of available data. Further supplementation of the results of this study is warranted in the future through the ongoing updating of online data; and (iii) the number of cases of strictly defined as vascular dementia and Parkinson’s disease is relatively low, so a more significant amount of GWAS pooled data is needed for future analysis.

## 5 Conclusion

Altogether, we confirmed a causal relationship between GM and dementia and its subtypes based on Mendelian randomization, including family Desulfovibrionaceae (id: 3169), family Lactobacillaceae (id: 1836), genus Ruminococcusgnavus group (id: 14376), genus Defluviitaleaceae UCG011 (id: 11287), genus Lactobacillus (id: 1837), order Desulfovibrionales (id: 3156), family Desulfovibrionaceae (id: 3169), genus Sellimonas (id: 14369), order Bacillales (id: 1674), order Desulfovibrionales (id: 3156), genus Lachnospiraceae NK4A136 group (id:11319), order Victivallales (id: 2254), class Alphaproteobacteria (id: 2379), genus Ruminococcusgnavus group (id: 14376), order Bacillales (id: 1674), genus Butyricimonas (id: 945), phylum Lentisphaerae (id: 2238), genus Ruminococcusgnavus group (id: 14376), genus Hungatella (id: 11306), genus Oscillibacter (id: 2063), and order Burkholderiales (id: 2874). These 21 GMs hold promise as novel markers for the future diagnosis of dementia and its subtypes, as well as new targets for therapy.

## Data availability statement

The original contributions presented in the study are included in the article/Supplementary material further inquiries can be directed to the corresponding author.

## Author contributions

JF: Methodology, Software, Visualization, Writing – original draft, Writing – review & editing. YQ: Formal analysis, Investigation, Visualization, Writing – original draft. LX: Funding acquisition, Project administration, Writing – review & editing. XD: Funding acquisition, Project administration, Resources, Writing – review & editing.

## Footnotes

^1^https://mibiogen.gcc.rug.nl

^2^https://r5.finngen.fi/

^3^https://www.ebi.ac.uk/gwas/home

^4^https://mrcieu.github.io/TwoSampleMR/

^5^https://github.com/rondolab/MR-PRESSO
