# Supplementary material for: Causal relationship between gut microflora and dementia: a Mendelian randomization study
Source: Front Microbiol. 2024 Jan 15;14:1306048. doi: 10.3389/fmicb.2023.1306048 (PMC10822966; doi:10.3389/fmicb.2023.1306048)
Supplement: Supplementary file 7 [file Table_7.DOCX]

Supplementary Material

## Supplementary Figures


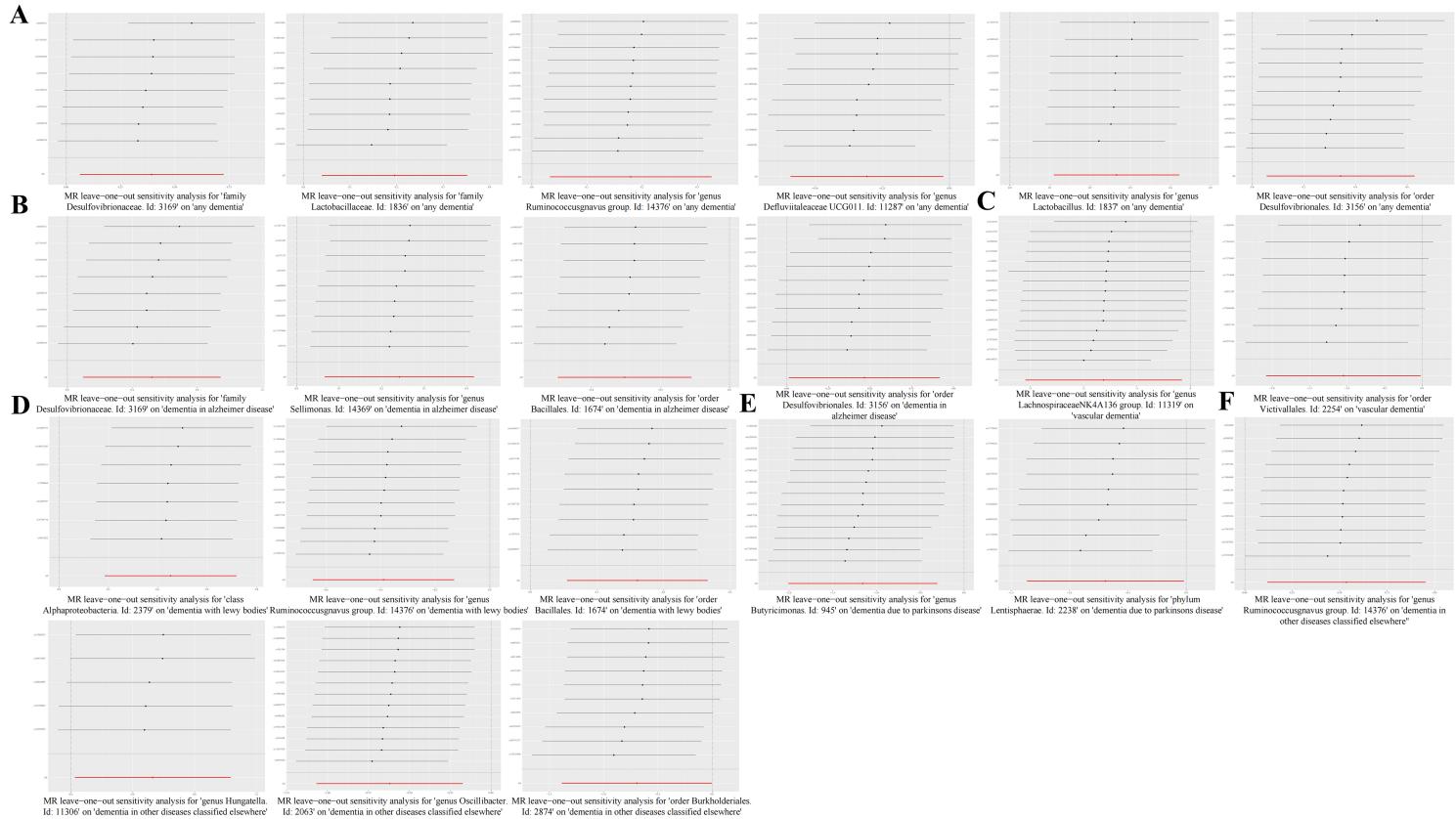


**Supplementary Figure 1.** Leave-one-out analysis for **(A)** 6 GM taxa on Any Dementia, **(B)** 4 GM taxa on Dementia in Alzheimer disease, **(C)** 2 GM taxa on Vascular dementia, **(D)** 3 GM taxa on Dementia with Lewy body, **(E)** 2 GM taxa on Parkinsonism dementia, and **(F)** 4 GM taxa on Dementia in other diseases classified elsewhere.

## Supplementary Tables

**Supplementary Table 1.** Detailed information of instrumental vatiables used in MR analyses.

**Supplementary Table 2.** Causal analysis of gut flora with dementia and its classification by five MR methods.

**Supplementary Table 3.** The heterogeneity results from the Cochran's Q test.

**Supplementary Table 4.** MR-PRESSO test results and MR-Egger intercept test results for the association between gut microbiota and dementia.

**Supplementary Table 5.** Reverse MR analysis results.

**Supplementary Table 6.** MR analysis results of the validation group.
