# Supplementary figures and images for: Causal relationship between gut microflora and dementia: a Mendelian randomization study
Source: Front Microbiol. 2024 Jan 15;14:1306048. doi: 10.3389/fmicb.2023.1306048 (PMC10822966; doi:10.3389/fmicb.2023.1306048)

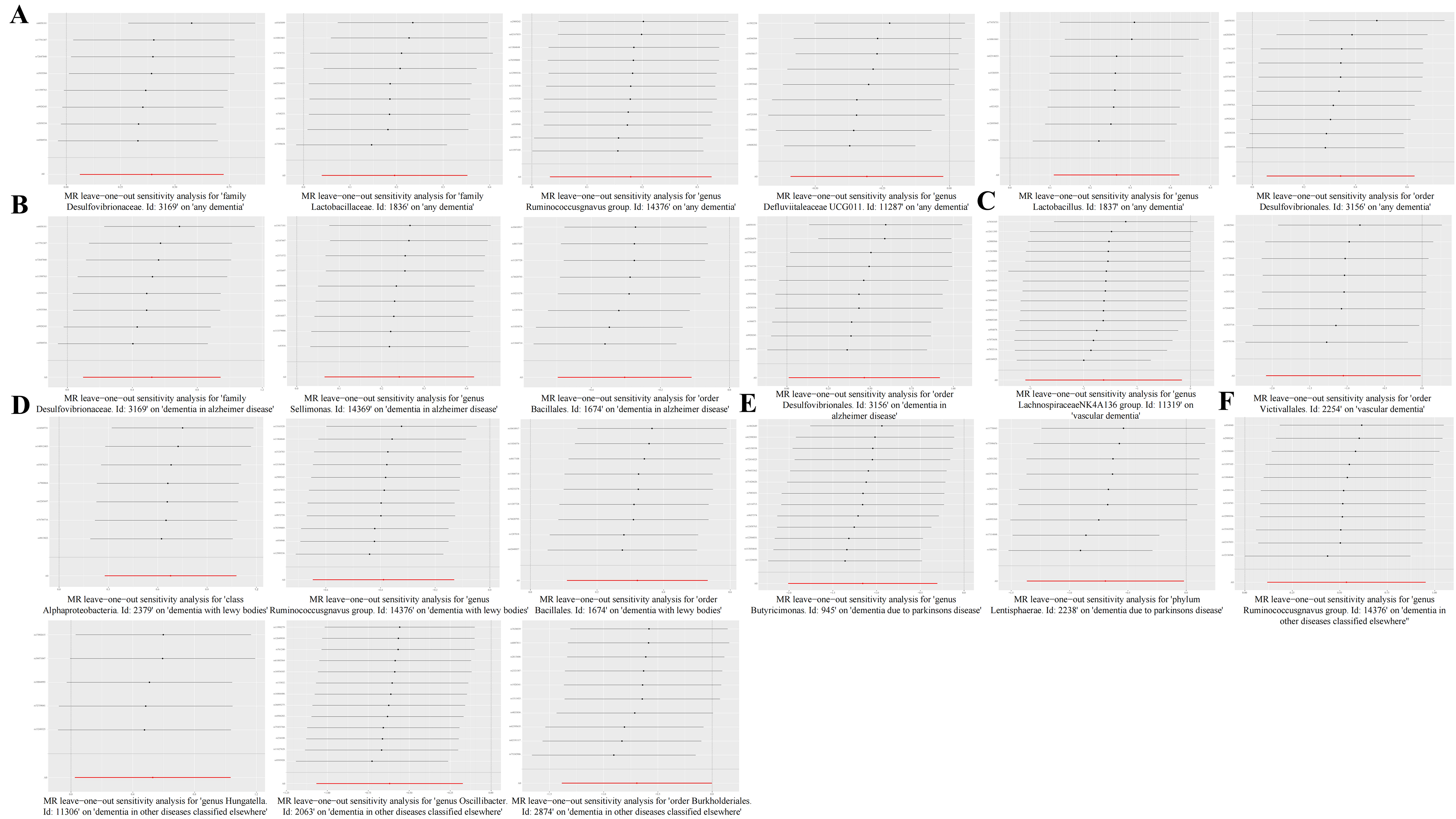

Supplement: Supplementary file 8 [file Image_1.JPEG]
